# Social Isolation and Acceptance of the Learning Management System (LMS) in the time of COVID-19 Pandemic: An Expansion of the UTAUT Model

**DOI:** 10.1177/0735633120960421

**Published:** 2021-04

**Authors:** Syed A. Raza, Wasim Qazi, Komal Akram Khan, Javeria Salam

**Affiliations:** 1 Department of Management Sciences, IQRA University, Karachi, Pakistan; 2 Department of Education and Learning Sciences, IQRA University, Karachi, Pakistan

**Keywords:** COVID-19, corona fear, social isolation, E-learning, learning management system (LMS), UTAUT, behavior, higher education, Smart-PLS

## Abstract

The COVID-19 Pandemic has led to social isolation; however, with the help of technology, education can continue through this tough time. Therefore, this research attempts to explore the Unified Theory of Acceptance and Use of Technology (UTAUT) through the expansion of the model. Also, make it relevant to investigate the influence of social isolation, and the moderating role of Corona fear on Behavioral Intention of the Learning Management System and its Use Behavior of Learning Management System among students. The data was analyzed using Partial Least Square (PLS) and Structural Equation Modelling (SEM). The findings show a positive link of Performance Expectancy (PE), Effort Expectancy (EE), Social Influence (SI), and Social Isolation on Behavioral Intention of LMS and, also between Behavioral Intention of LMS and its Use behavior. Moreover, the results of the moderation analysis show that Corona fears only moderates the link of Performance Expectancy and Social influence with Behavioral Intention of LMS. The findings imply the need for improving the LMS experience to increase its Behavioral Intention among students. Finally, the author's recommendation for future researchers is to examine the extended model in other countries and territories to analyze Coronavirus's influence on e-learning acceptance.

## Introduction

The COVID-19 emerged in the year 2019, in the Wuhan, China, and was soon declared a pandemic as it spread worldwide due to its extremely high infectious rate. According to the World Health Organization (2020) report, over 130 countries and territories had confirmed the presence of Coronavirus as its cases emerged during the mid of March 2020. As the highly-infectious disease has a relatively high mortality rate, it has caused an increase of fear among the people naturally (Ahorsu et al., 2020), as the worry regarding the COVID-19 was based on contact with individuals who might be infected with the disease (Lin, 2020).

As a response to the COVID-19 crisis, governments around the world have issued public policies that include social distancing, isolation, and self-quarantine (Anderson et al., 2020), having unprecedented economic and psychosocial consequences worldwide. While millions of people around the world stay in their homes to prevent the Coronavirus from spreading, their livelihoods have been obstructed, and, in the students' case, their access to education has been impeded. However, as countries go into lockdown, the advancement in information technology gives light to possible alternatives. The dramatic changes caused by the evolution of Information technology in all aspects of life, especially considering its involvement, higher education is crucial to discuss during the COVID-19 pandemic. Technology has always helped enhance the simplest of tasks, such as the advancement of the traditional learning process. A technology that lies under the umbrella of e-learning has made it possible to continue the learning process during the lockdown (Zwain, 2019). This technology is referred to as the Learning Management System (LMS). LMS is defined by Alias and Zainuddin (2005), as a web-based technology developed to improve the learning process through its proper planning, application, and evaluation in educational institutions. Using LMS in the learning process helps facilitate e-learning as it provides educational material without the constraint of time or place, (Ain et al., 2016), enabling students and teachers to interact via the internet and facilitates sharing of course-related information and resources (Al-Busaidi & Al-Shihi, 2010; Lonn et al., 2011). This indicates that the use of this technology during the COVID-19 pandemic is the need of the hour to keep the learning process continued. A few examples of LMS used in educational institutions include Moodle, WebCT, Blackboard, and Desire2Learn (Iqbal, 2011; Waheed et al., 2016). Hassanzadeh et al. (2012) revealed in his study that, with the advent of information technology, the definition of higher education had been changed. Therefore, the area of technology acceptance was seen by scholars as a mature area in the role of information systems in science (Venkatesh et al., 2003). According to Teo (2011), technology acceptance is the willingness of an individual to adopt the use of technology for facilitating task performance based on the support it was designed to provide. In recent times, the acceptance of e-learning systems and technologies is being investigated by researchers in different educational environments around the world, using different models based on distinct criteria (Decman, 2015; Raza, Khan, & Rafi, 2020). Considering the context of the higher education sector, it is crucial to investigate factors that result in e-learning technology acceptance among students, as investing e-learning systems requires huge investment in resources and infrastructure (Ma & Yuen, 2011). If the students do not accept the new system of learning, the return on investment of universities would be reduced (Zwain, 2019). The existing literature revealed that the acceptance of LMS among students in higher education varies from country to country (Zwain, 2019), as Arab universities in the Middle Eastern region registered level of e-learning acceptance was low (Matar et al., 2011) while a high-acceptance rate of the e-learning system was registered in western countries (Decman, 2015).

The present paper investigates the factors that influence the acceptance of LMS from the perspective of students during the COVID-19 pandemic. For this purpose, the theory of UTAUT is considered the well-developed, updated, and relevant technology acceptance theory by researchers, as it has been merged from existing recognized theories of technology acceptance (Decman, 2015). The reason for the development of the UTAUT model was to explore a unified view of Information technology (Venkatesh et al., 2003). The model has been subsequently validated by Venkatesh et al. (2003) in a longitudinal research study, where it was found that the model accounted for 70 percent variance in BI to use technology and 50 percent about its actual use. Therefore, this theory was chosen among the other theories as it is more comprehensive, enabling higher explanatory power than the early theories used for studying technology acceptance. Through the years, researchers have explored the model through the incorporation of several factors to understand technology acceptance relevant to the situational factors of the area being researched. Lin and Anol (2008) added online social support to understand its influence on the use of network information technology in Taiwan. Further, Raza et al. (2019) studied the factors which affect mobile banking (M-banking) acceptance in Islamic banks of Pakistan by using the modified uniﬁed theory of acceptance and use of technology (UTAUT) model. Moreover, Chao (2019) aimed to empirically test the factors that influence student's BI towards mobile learning through the addition of different factors such as perceived enjoyment, satisfaction, trust, risk, and mobile self-efficacy. Taiwo and Downe (2013) and Dwivedi et al. (2011) revealed, in their recent meta-analysis of the outcomes of the UTAUT studies, that its constructs are positively and significantly associated with the existing literature, but stress the lack of investigation of a moderator in several studies. Therefore, the extension of this model through the incorporation of Social factors and the Corona Fear will help understand the user's behavioral intention of technology acceptance in light of the recent pandemic and its subsequent behavioral use. Testing the acceptance of e-learning system using the UTAUT model is sufficient as it is the latest, most up to date technology acceptance theory that is widely recognized by scholars (Decman, 2015). The findings of the extended model will prove to be useful for understanding the acceptance of LMS among students, in this way, educational institutions will focus on the system's effective implementation and invest in e-learning technology for a good purpose.

This research paper follows the introduction with the literature review, elaborating on the theoretical background and the hypotheses development that is to be tested. Then, the paper emphasizes the research methodology used for measuring the impact of variables, and the used sampling and data collection methods. Then, the data analysis techniques and findings have been discussed. Lastly, the paper is concluded with the implications of the findings and future research directions that follow the study's limitations.

## Literature Review

### Theoretical Background

This paper develops an integrated model through the extension of the Unified Theory of Acceptance and Use of Technology (UTAUT) by adding the independent variable Social Isolation that is caused by the recent COVID-19 pandemic, and the moderating variable that is Corona Fear, to the model's pre-existing constructs that include Performance Expectancy (PE), Effort Expectancy (EE), Social Influence (SI) and Facilitating Conditions (FC). The original UTAUT was introduced by Venkatesh et al. (2003). He reviewed eight existing theories to develop a unified model. The theories include the Theory of Reasoned Action (TRA), Innovation Diffusion Theory (IDT), Social Cognitive Theory (SCT), Technology Acceptance Model (TAM), Theory of Planned Behavior (TPB), Model of PC Utilization (MPCU), Motivational Model (MM), and, the Combined TAM and TPB (C-TAM-TPB). The integrated model enables scholars to view and show the complete picture of the predictors of technology acceptance, according to Al-Imarah et al. (2013). Venkatesh et al. (2003) revealed that his unified model predicts 69 percent variance in Behavioral Intention of users, which is higher than the pre-existing models that only predicted 17 to 53 percent. Therefore, this model is a useful tool for investigating student's acceptance of the LMS during the COVID-19 pandemic. Thus, the authors have deployed the model and extended it to assess the role of Social Isolation on Behavioral Intention of LMS and the moderating effect of Corona Fear caused by the Pandemic

### Hypotheses Development

#### Performance Expectancy (PE)

The extent of an individual's perception regarding technology's usefulness to perform different tasks is called Performance Expectancy (PE) (Venkatesh et al., 2003; Ain et al., 2016), and in the case of evaluating the acceptance of LMS among the students, it is regarded as the student's belief regarding the effectiveness of the system for studying (Decman, 2015). Lwoga and Komba (2015) defining is to what extent students understand the system's potential to allow them to perform better in their classes. It suggests that consumers would be able to implement the technology if their understanding is that their efficiency benefits from it. The use of a learning management system will enable students to use technology for their educational activities. Several scholars have used the voluntary and mandatory setting to evaluate the influence of a technology's PE on the Behavioral Intention of using it and found a significant direct effect (Casey & Wilson-Evered, 2012; Dwivedi et al., 2011; Gupta et al., 2008; ŠUmak et al., 2011; Venkatesh et al., 2003; Zhou et al., 2010). In the area of the e-learning environment, Sumak et al. (2010) analyzed the impact of PE on the BI of LMS and found that it is positively significant. Thus, the existing literature shows that student's belief that using LMS would improve their Performance will enable them to adopt its use readily. Therefore, based on the review of the literature, the following hypothesis is proposed by the authors:

*H1: PE positively influences BI of LMS*

#### Effort Expectancy (EE)

Yoo et al. (2012), revealed that the most influential factors of the UTAUT model are the Effort Expectancy (EE) which is considered as an intrinsic element, as it is the amount of effort an individual perceives to invest to use a technology, which is low in general due to the user-friendly nature of information technology (Decman, 2015). Researchers assess the relationship between Effort expectancy during the early stages of adoption of technology, where they found it had a direct impact on BI (Gupta et al., 2008; Venkatesh et al., 2003), while Venkatesh (2000) revealed that it becomes insignificant over time and Gruzd et al. (2012) found a negative relationship. Raman and Don (2013) discussed that the relationship of EE on BI was positively significant when they tested the variables in the context of pre-school teachers' acceptance of LMS. It is assumed by the authors of this paper that student's belief regarding the low degree of effort required to use LMS that leads to a higher BI of using it. Therefore, based on the review of the literature, the following hypothesis is proposed by the authors:

*H2: EE positively influences the BI of LMS.*

#### Social Influence (SI)

Social influence (SI) constitutes the reflection of peers, instructors, and friends' perceptions regarding technology on the individual's Behavioral intentions within a social environment (Venkatesh et al., 2003). While evaluating the acceptance of LMS, SI is the degree of a student's social circle influencing their BI of LMS. As information technology has advanced and social networking sites are emerging, the focus of this factor has shifted from physical to virtual (Decman, 2015). Researchers have found a direct relationship of SI on BI of individuals regarding the use of technology in both voluntary and mandatory settings (Gruzd et al., 2012; Gupta et al., 2008; Venkatesh et al., 2003). An international study was done by Im et al. (2011), revealed that the role Social influence played on BI was positively significant and relatively higher in Korean respondents compared with the US. Another scholar declared that employees were socially influenced to adopt the use of services offered by e-government (Al-Shafi et al., 2009). Investigating the factors concerning the area e-learning system Fidani and Idrizi (2012) found that there was a positive influence of social influence on student's BI to use LMS. Therefore, based on the review of the literature, the following hypothesis is proposed by the authors:

*H3: SI positively influences the BI of LMS.*

#### Facilitating Conditions (FC)

Venkatesh et al. (2003) refer to Facilitating Conditions (FC) as the availability of adequate support and resources for the proper use of technology. In the context of the E-learning environment, FC focuses on the accessibility of technical and organizational infrastructure for the adoption and use of the LMS. This includes training, technical support, and the required infrastructure (Decman, 2015). The original model of UTAUT found that the role FC had on an individual's BI to use a particular technology was direct but insignificant (Venkatesh et al., 2003). While Dwivedi et al. (2011) revealed that the link of FC conditions was found to be the least significant with BI among the four factors of the UTAUT model. According to Nanayakkara (2007), lack of assistance and timely support due to limited availability of resources and information will hinder the acceptance level of web-based technology among students, because they need their teacher's and technical support to positively influence their use of Learning Management System (Ain et al., 2016). The existing literature shows that the e-learning acceptance level is positively influenced by facilitating conditions (Bakar et al., 2013). This indicates student's perception regarding the availability of facilitating conditions is a valid predictor of their BI of LMS. Therefore, based on the review of the literature, the following hypothesis is proposed by the authors:

*H4: FC positively influences the BI of LMS.*

#### Social Isolation

De Jong Gierveld et al. (2016) define social isolation as an individual's absence or the low number of meaningful ties with other people, thus making them socially isolated. The COVID-19 Pandemic has forced countries to go into lockdown, and drastically reduced social gatherings, through the encouragement of social distancing as it is required to eradicate the spread of Coronavirus. Due to the closing of classrooms, public markets and the postponement and cancellation of activities and meeting, social distancing decreases social contact between people in the group, leading to isolation around the world (Wilder-Smith & Freedman, 2020), the authors predict socially isolated students will be positively stimulated to take online classes through Learning Management System. Therefore, based on this assumption, the following hypothesis is proposed by the authors:

*H5: Social Isolation positively influences the BI of LMS.*

#### Behavioural Intention (BI) of LMS

A person's intention to adopt the use of a specific technology for performing various tasks is referred to have his or her Behavioral Intention (BI) (Ain et al., 2016). Ngai et al. (2007) defined BI as the level of commitment a person shows to engage in a specific behavior, which in the context of this paper is the student's commitment level for accepting the use of LMS to fulfill their educational course objectives. Several scholars have analyzed the role of BI of technology on its actual use behavior and found that there is a direct and significant link (Davis, 1989; Motaghian et al., 2013; Raman & Don, 2013; Wang & Wang, 2009). Nicholas-Omoregbe et al. (2017) revealed in a paper that the BI of students regarding the adoption of the e-learning system has a positive link with their use behavior, which ultimately results in better grades. Use Behavior is the extent of the actual use of technology by an individual to perform various tasks (Bagozzi, 1981). In harmony with the existing literature, the authors of this study, expect a positive association between Behavioral intention of Learning Management System and its Use Behavior. Therefore, based on the review of the literature, the following hypothesis is proposed by the authors:

*H6: BI of LMS positively affects the Use Behavior of LMS.*

#### Moderating Effect of Corona Fear

Mertens et al. (2020) define fear as an adaptive emotion that mobilizes energy in an individual to deal with a potential threat. Pakpour and Griffiths (2020) revealed that unexpected and extraordinary situations such as disease outbreaks could cause fear among people, and therefore it is one of the psychological aspects of the COVID-19 pandemic. This indicates the need to determine its effect on students, especially concerning acceptance and use of LMS that is being deployed by educational institutions, to continue the learning process. So, the authors of this paper propose the following hypotheses for analyzing the moderating role of Corona Fear:

*H7: Corona Fear moderates the relationship between PE and the BI of LMS.*

*H8: Corona Fear moderates the relationship between EE and the BI of LMS.*

*H9: Corona Fear moderates the relationship between SI and the BI of LMS.*

*H10: Corona Fear moderates the relationship between FC and the BI of LMS.*

*H11: Corona Fear moderates the relationship between Social Isolation and the BI of LMS.*

## Research Methodology

### Research Model

**Figure 1.**
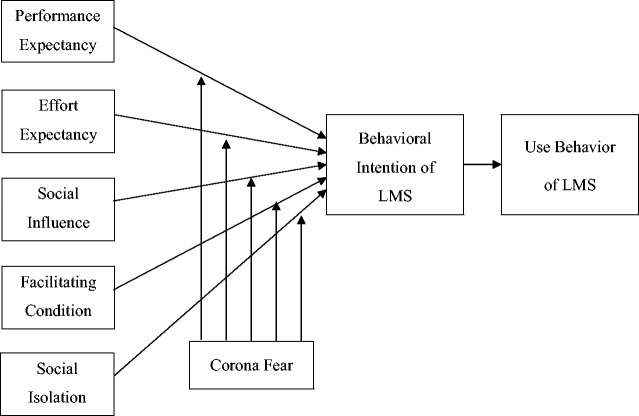
Conceptual Model.

Since the original UTAUT model by Venkatesh et al. (2003) offered relevant factors for determining student's behavioral intention towards LMS and its Use behavior, they were used for fulfilling the purpose of this research. However, the model needed to be extended to explore the acceptance of LMS among students enrolled in higher-education institutions, during the COVID-19 pandemic. For this reason, social isolation was added as an independent variable, while Corona's fear was included as a moderating variable (Figure 1).

### Data Collection and Instrumentation

The sample used in this research included students enrolled in the Universities of Karachi, Pakistan. For the development of the scale for data collection, items were adapted from the existing literature. The items for measuring variables were adapted from Venkatesh et al. (2003), Zwain (2019), and Garone et al. (2019). The scale for measuring constructs was based on a five-point Likert scale design and consisted of a total of 35 items. Responses for analysis were collected from students by distributing the questionnaire online. The sample size selected for the data was based on the guidelines presented by Raza and Hanif (2013), Comrey and Lee (2013), Raza et al. (2020), Sharif and Raza (2017) that the sample of 50 is considered as poor, 300 as good, 500 as very good and 1000 was considered as an excellent sample with respect to factor analysis. Hence, we gathered a total of 516 responses.

### Demographics

The demographic analysis showed the following description of the respondent's profiles, as depicted in Table 1. The gender distribution of the respondents showed that male respondents at 54.8 percent comprised the majority, while female respondents totaled 45.2 percent. While concerning the ages of the respondents, the analysis showed that 5.6 percent were less than or equal to 19 years old, 30.8 percent were between 20-24, 38.4 percent were between 25-29, 18.4 percent were between 30-34, 5.8 percent were between 35-39, and lastly only 1 percent were more than and equal to 40 years old. Moreover, analyzing the level of education of the respondents, it was found that Graduates constituted the majority totaling 57.6 percent, 33.1 percent were undergraduates, 9.1 percent were postgraduates, and lastly, only 0.2 percent of respondents marked other.

**Table 1.** Respondent’s Profile (N = 516).

Demographic items	Frequency	Percentile
Gender		
Male	283	54.8%
Female	233	45.2%
Age		
Less than and equal to 19	29	5.6%
20–24	159	30.8%
25–29	198	38.4%
30–34	95	18.4%
35–39	30	5.8%
More than and equal to 40	5	1.0%
Education		
Undergraduate	171	33.1%
Graduate	297	57.6%
Post graduates	47	9.1%
Other	1	0.2%

Source: Author’s estimation.

## Data Analysis and Results

In the present study, the partial least square structural equation modeling (PLS-SEM) technique was applied to the data, using Smart PLS version 3.2.3 (Ringle et al., 2015). The criteria of Raza et al. (2020) has been followed in the present research. Hence, we applied a bootstrapping method with 5000 subsamples to determine the significance value for each path coefficient. PLS-SEM was performed in two steps. The first step involves the evaluation of the measurement model; the second step involves the evaluation of the structural model. In the measurement model, we assessed the construct validity and discriminant validity criteria, whereas, in the structural model, we assessed the R2 and the significance of the path coefficients.

Ringle et al. (2005) revealed that Structural Equation Modeling (SEM) is a valid statistical technique that helps analyze the validity of a study's theory using statistical facts and figures. The current paper deploys a variance-based method for analyzing the hypothetical model. The execution of PLS-SEM using Smart PLS software is a suitable method for analyzing and examining several integrated models in various contexts for research (Chin, 1998; Henseler et al., 2009).

### Measurement Model

A measurement model assesses the scale's competency that is used for research purposes, which can be determined through obtaining Composite Reliability (CR), Individual Item Reliability (IIR), Convergent Validity (CV), and the Average Variance Extracted (AVE).

The value that determines the scale's reliability is referred to as the Cronbach's alpha, the criteria of which is given by Tabachnick and Fidell (2007) that is greater than 0.55. Since the values meet the prescribed criteria, the scale is determined to be reliable. Straub (1989) said that the value of Composite reliability should be greater than 0.7, and the values in Table 2 show that the said criteria have also been met.

**Table 2.** Measurement Model Results.

	Items	Loadings	Mean	Standard deviations	Cronbach’s alpha	Composite reliability	Average variance extracted
BI	BI1	0.756	3.840	1.007			
	BI2	0.844	3.740	0.964	0.808	0.874	0.635
	BI3	0.817	3.920	1.020			
	BI4	0.768	3.860	0.944			
CF	CF1	0.824	3.980	0.794			
	CF2	0.845	3.940	0.860			
	CF3	0.809	3.910	0.847	0.912	0.932	0.696
	CF4	0.872	3.900	0.867			
	CF5	0.822	3.990	0.844			
	CF6	0.830	3.934	0.928			
EE	EE1	0.877	3.770	1.094			
	EE2	0.855	3.800	1.016	0.826	0.896	0.742
	EE3	0.852	3.970	1.035			
FC	FC1	0.785	3.850	0.992			
	FC2	0.887	3.910	0.980	0.874	0.914	0.726
	FC3	0.869	3.870	0.949			
	FC4	0.866	3.860	0.980			
PE	PE1	0.821	4.160	0.923			
	PE2	0.851	4.100	0.998	0.846	0.897	0.685
	PE3	0.856	4.270	0.901			
	PE4	0.780	4.140	0.939			
SI	SI1	0.746	3.840	0.981			
	SI2	0.826	3.710	1.001	0.814	0.878	0.643
	SI3	0.801	3.820	0.972			
	SI4	0.830	3.820	1.023			
SIS	SIS1	0.881	4.050	0.761			
	SIS2	0.880	4.230	0.666			
	SIS3	0.934	4.210	0.654	0.933	0.949	0.788
	SIS4	0.882	4.200	0.692			
	SIS5	0.859	4.250	0.651			
UB	UB1	0.758	3.840	1.066			
	UB2	0.858	3.840	1.041			
	UB3	0.827	3.770	1.045	0.863	0.901	0.647
	UB4	0.826	3.780	1.034			
	UB5	0.747	3.930	0.909			

*Notes: BI = Behavioral Intention of LMS, CF = Corona Fear, EE = Effort Expectancy, FC =  Facilitating Conditions, PE = Performance Expectancy, SI = Social Influence, SIS = Social Isolation, UB =  Use Behavior of LMS.*

Moreover, the benchmark for individual reliability is that it should be greater than 0.7 (Churchill, 1979), which can be seen in Table 2. Since all the loadings have a value that is greater than the benchmark, hence it is deemed reliable for research. Furthermore, the analysis shows that every variable has a higher than 0.5 Average Variance Extract (AVE), which meets the criteria given by Fornell and Larker (1981). Hence, the scale's convergent validity is confirmed.

Moving on to the analysis of discriminant validity, the values of which are displayed in Table 3, it can be seen that the discriminant validity has been measured by performing a cross-loading analysis and extracting the AVE. Its criteria have been determined by Fornell and Larker (1981) that says that the value of AVE should be higher than the correlation of the variables, and it can be seen in Table 3 that it has been met as the diagonally represented values of the square root of AVE satisfy the given criteria.

**Table 3.** Fornell-Larcker Criterion.

	BI	CF	EE	FC	PE	SI	SIS	UB
BI	**0.797**							
CF	0.561	**0.834**						
EE	0.558	0.609	**0.861**					
FC	0.410	0.474	0.328	**0.852**				
PE	0.375	0.266	0.327	0.306	**0.828**			
SI	0.634	0.627	0.683	0.437	0.396	**0.802**		
SIS	0.279	0.402	0.231	0.425	0.041	0.243	**0.888**	
UB	0.537	0.566	0.431	0.763	0.390	0.551	0.247	**0.804**

*Notes: *BI = Behavioral Intention of LMS, CF = Corona Fear, EE = Effort Expectancy, FC =  Facilitating Conditions, PE = Performance Expectancy, SI = Social Influence, SIS = Social Isolation, UB =  Use Behavior of LMS. The diagonal elements (bold) represent the square root of average variance extracted (AVE).

Furthermore, Table 4 depicts the cross-loadings of the items, and results reveal that all the items are loaded higher in their relevant construct in comparison with the corresponding variable. Moreover, the cross-loading difference is also higher than the suggested threshold of 0.1(Gefen & Straub, 2005).

**Table 4.** Loadings and Cross Loadings.

		BI	CF	EE	FC	PE	SI	SIS	UB
BI1	I intend to continue using LMS	**0.756**	0.459	0.387	0.278	0.221	0.470	0.197	0.406
BI2	For my studies, I would use LMS	**0.844**	0.480	0.492	0.328	0.262	0.521	0.236	0.442
BI3	I will continue to use LMS on a regular basis	**0.817**	0.444	0.418	0.371	0.407	0.521	0.245	0.454
BI4	Because of the possibilities that LMS offers, I plan to approach my next course more effectively	**0.768**	0.408	0.481	0.327	0.296	0.506	0.209	0.410
CF1	I do not want to leave the house because of the risk of getting infected by COVID 19 pandemic	0.485	**0.824**	0.506	0.409	0.238	0.549	0.314	0.469
CF2	I am concerned that I may get sick from COVID-19 pandemic during the next 6 months	0.468	**0.845**	0.524	0.411	0.240	0.528	0.347	0.499
CF3	I am feeling anxious about COVID-19 pandemic	0.434	**0.809**	0.514	0.409	0.185	0.506	0.279	0.462
CF4	I am concerned that someone in my immediate family may get sick from COVID-19 pandemic during the next 6 months	0.492	**0.872**	0.526	0.407	0.185	0.526	0.371	0.474
CF5	I am scared about getting infected by COVID-19 pandemic	0.409	**0.822**	0.481	0.351	0.236	0.498	0.311	0.439
CF6	I see the possibility that Covid-19 pandemic will break out in the area where	0.507	**0.830**	0.496	0.379	0.248	0.529	0.377	0.484
EE1	I live and work	0.461	0.487	**0.877**	0.247	0.224	0.569	0.196	0.339
EE2	Learning how to use LMS is easy for me.	0.480	0.554	**0.855**	0.262	0.327	0.606	0.162	0.363
EE3	I find the system to be flexible to interact with.	0.499	0.530	**0.852**	0.335	0.291	0.588	0.236	0.409
FC1	I have resources to use LMS	0.328	0.509	0.342	**0.785**	0.263	0.389	0.244	0.665
FC2	I have the knowledge to use LMS	0.359	0.399	0.272	**0.887**	0.303	0.365	0.411	0.667
FC3	A specific person (or group) is available to assist when difficulties arise with LMS	0.337	0.347	0.252	**0.869**	0.228	0.346	0.391	0.629
FC4	Using the system fits into my study styles.	0.373	0.367	0.259	**0.866**	0.249	0.390	0.395	0.642
PE1	I find LMS useful for studies.	0.295	0.182	0.333	0.234	**0.821**	0.310	0.038	0.308
PE2	LMS allows me to accomplish class activities more quickly	0.339	0.204	0.222	0.286	**0.851**	0.335	0.032	0.354
PE3	LMS increases learning productivity	0.288	0.215	0.284	0.227	**0.856**	0.335	0.024	0.295
PE4	Using the system would make it easier to do my studies	0.312	0.279	0.251	0.259	**0.780**	0.328	0.039	0.326
SI1	My peers who influence my behavior think that I should use LMS.	0.473	0.512	0.444	0.332	0.203	**0.746**	0.175	0.460
SI2	My friends who are important to me think that I should use LMS.	0.518	0.477	0.507	0.353	0.322	**0.826**	0.171	0.433
SI3	Instructors whose opinions that I value prefer that I should use LMS.	0.487	0.499	0.579	0.383	0.383	**0.801**	0.247	0.477
SI4	I use the system because of the proportion of classmates who use the system	0.550	0.525	0.648	0.337	0.354	**0.830**	0.189	0.405
SIS1	I felt alone and friendless.	0.239	0.349	0.197	0.397	0.075	0.203	**0.881**	0.237
SIS2	I felt isolated from other people	0.230	0.326	0.181	0.371	0.000	0.192	**0.880**	0.180
SIS3	I have someone to share my feelings with	0.251	0.377	0.193	0.395	0.006	0.212	**0.934**	0.220
SIS4	I found it easy to get in touch with others when I needed others to felt they had to help me.	0.223	0.348	0.171	0.340	0.026	0.193	**0.882**	0.189
SIS5	When with other people, I feel separate from them.	0.284	0.376	0.266	0.378	0.067	0.265	**0.859**	0.260
UB1	I use LMS frequently during my academic period	0.362	0.449	0.323	0.671	0.332	0.398	0.197	**0.758**
UB2	I use many functions of LMS (e.g., discussion forum, chat session, messaging, download course contents, upload assignments, etc.	0.446	0.463	0.342	0.709	0.387	0.458	0.217	**0.858**
UB3	I depend on LMS	0.404	0.403	0.331	0.651	0.312	0.426	0.173	**0.827**
UB4	Use of LMS by our university is a good idea	0.406	0.400	0.333	0.669	0.332	0.456	0.159	**0.826**
UB5	LMS makes learning more interesting for the students	0.509	0.532	0.387	0.410	0.220	0.461	0.235	**0.747**

*Notes: *BI = Behavioral Intention of LMS, CF = Corona Fear, EE = Effort Expectancy, FC = Facilitating Conditions, PE = Performance Expectancy, SI = Social Influence, SIS = Social Isolation, UB = Use Behavior of LMS. All self-loading is significant (bold).

Finally, Table 5 shows the heterotrait-monotrait ratio of correlations (HTMT), the values of which are less than 0.85, confirming the validity, as per the criteria given (Henseler et al., 2015; Raza et al., 2018, 2020).

**Table 5.** Heterotrait-Monotrait Ratio (HTMT).

	BI	CF	EE	FC	PE	SI	SIS	UB
BI								
CF	0.652							
EE	0.682	0.700						
FC	0.487	0.533	0.387					
PE	0.448	0.303	0.393	0.354				
SI	0.780	0.728	0.828	0.520	0.474			
SIS	0.318	0.432	0.258	0.467	0.046	0.276		
UB	0.633	0.629	0.504	0.892	0.458	0.656	0.269	

*Notes: *BI = Behavioral Intention of LMS, CF = Corona Fear, EE = Effort Expectancy, FC =  Facilitating Conditions, PE = Performance Expectancy, SI = Social Influence, SIS = Social Isolation, UB =  Use Behavior of LMS.

Ultimately, as the scale's reliability and validity have been established through analyzing the measurement model, the distinctiveness of the framework has been confirmed, deeming it reliable and valid for moving forward to the analysis of the structural model.

### Structural Model

**Figure 2.**
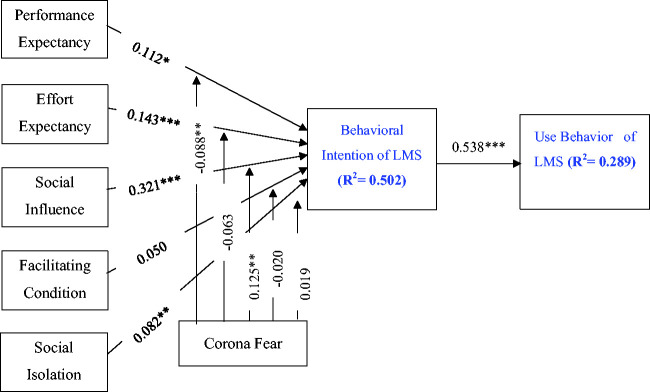
Results of Path Analysis.

The structural model analysis was done using standardized paths to get the results. Each path that has been tested in the structural model corresponds to the hypotheses developed by the authors of this research. The First-order analysis results are shown in Table 6, while the results of the moderating variable that have been tested are shown in Table 6. Moreover, Figure 2 depicts the results of Standardized Regression Weight (SRW). Also, the value of R-squared is mentioned in the model. R-squared is a goodness-of-fit measure for linear regression models. It is also termed as the coefficient of determination. This statistic indicates the percentage of the variance in the dependent variable that the independent variables explain collectively. The R^2^ for “Behavioral Intention of LMS” is 0.502, implying that 50.2% of the behavioral intention to use LMS is due to the latent variable in the model. Similarly, R^2^ for “Use Behavior of LMS” is 0.289, implying that 28.9% of the use behavior of LMS is because of the behavioral intention to use LMS.

**Table 6.** Results of Path Analysis-First Order and Moderating Role of Corona Fear.

Hypothesis	Regression path	Effect type	SRW	Remarks
*A: Results of path analysis-first order*				
H1	PE −> BI	Direct effect	0.112*	Supported
H2	EE −> BI	Direct effect	0.143***	Supported
H3	SI −> BI	Direct effect	0.321***	Supported
H4	FC −> BI	Direct effect	0.050	Not supported
H5	SIS −> BI	Direct effect	0.082**	Supported
H6	BI −> UB	Direct effect	0.538***	Supported
*B: Moderating role of corona fear*				
H7	PE −> BI	Indirect effect	−0.088**	Supported
H8	EE −> BI	Indirect effect	−0.063	Not supported
H9	SI −> BI	Indirect effect	0.125**	Supported
H10	FC −> BI	Indirect effect	−0.020	Not supported
H11	SIS −> BI	Indirect effect	0.019	Not supported

*Notes: *BI = Behavioral Intention of LMS, CF = Corona Fear, EE = Effort Expectancy, FC = Facilitating Conditions, PE = Performance Expectancy, SI = Social Influence, SIS = Social Isolation, UB = Use Behavior of LMS, SRW=Standardized regression weight.***p < 0.01, **p < 0.05, *p < 0.10.

### Discussion

Table 6 depicts the findings of the first-order analysis. The hypotheses tested have shown that the relationship between PE (B=0.112 p < 0.1), EE (B=0.143 p < 0.01), and SI (B=0.321 p < 0.01) is found to be positively significant with BI of LMS. Therefore, the H1, H2, and H3 have been accepted. This means that students' BI to use LMS in Pakistani Universities is influenced by the expectation of its usefulness, the effort required to invest in its use, and also social influence. These results are inconsistent with the study of Zwain (2019), who examined the variables in the University of Kufa in Iraq by collecting data from faculty and respondents and found that PE and EE did not have a positive influence, while SI did on the BI to use LMS. While Decman (2015) revealed that the link between PE and SI on BI to use e-learning technology was significantly positive; however, his research did not support the positive influence of EE. Meanwhile, other past studies in the literature show positive relationships among the variables making the results consistent with their findings (Casey & Wilson-Evered, 2012; Dwivedi et al., 2011; Gupta et al., 2008; ŠUmak et al., 2011; Venkatesh et al., 2003; Zhou et al., 2010)

Social Isolation (B = 0.082 p < 0.05), the extended variable in the UTAUT model, has been found to have a positive and significant relationship with BI of LMS. Thus, H5 has also been accepted, indicating that socially isolated students would be inclined towards using LMS for gaining knowledge and completed course objectives.

In contrast, FC (B = 0.050 p > 0.1) were found to have a positive but insignificant relationship with BI of LMS. Hence, H4 has been rejected. This means that the availability of timely assistance and the necessary infrastructure does not affect the intention of the student regarding BI to use LMS for completion of their course work. The existing literature showed that there was a direct but insignificant relationship between FC and BI to use LMS (Dwivedi et al., 2011; Venkatesh et al., 2003). It indicates students aren't pleased with the support. This can also be argued that students are reluctant to embrace technology and, therefore, not satisfied with the assistance because their crucial concern is weak internet connectivity.

Moreover, the hypothesis of BI of LMS (B = 0.538 p < 0.01) was also found to be positively linked with the Use Behavior of LMS. Therefore, H6 has been accepted. This means that students who have a higher level of BI to use LMS will be positively influenced towards actually using LMS. This finding is consistent with the studies of Davis (1989), Wang and Wang (2009), Motaghian et al. (2013) and Raman and Don (2013), they concluded that there is a significantly positive link between BI and actual use.

Moving on to the results of the moderating variable, shown in Table 6, the hypotheses concerning the Corona fear as a moderator was found to be moderating the link of PE and SI on BI of LMS was found to be positive. Thus, H7 and H9 were supported. This means that the presence of Corona fears among students during the pandemic does strengthen the association of PE and SI with BI to use LMS. However, the findings showed that Corona's fear did not have a moderating role in the relationship of EE, FC, and Social Isolation with BI of LMS. Hence, H8, H10, and H11 are rejected. This means that the presence of Corona's fear among students does not strengthen the relationship among the variables.

### Interpretation of Tables 7, 8 and 9

Lastly, Tables 7, 8, and 9 represent the results of Stone-Geisser or O2, SRMR indicator, and f2 coefficients. The Stone-Geisser or O2 evaluates the predictive relevance of each of the model's endogenous variables. The next table displays the results of the SRMR indicator, and it estimates the goodness of fit of the structural model. The last table depicts the results of f2 coefficients that analyze the effect size of the relationships between variables.

**Table 7.** Stone-Geisser or O2.

	Q² (=1-SSE/SSO)
BI	0.293
CF	
EE	
FC	
PE	
SI	
SISO	
UB	0.170

**Table 8.** SRMR Indicator.

	Saturated model	Estimated model
SRMR	0.060	0.104

**Table 9.** F-square.

	BI	CF	EE	FC	PE	SI	SISO	UB
BI	** **	** **	** **	** **	** **	** **	** **	0.406
CF	0.031	** **	** **	** **	** **	** **	** **	** **
EE	0.016	** **	** **	** **	** **	** **	** **	** **
FC	0.003	** **	** **	** **	** **	** **	** **	** **
PE	0.017	** **	** **	** **	** **	** **	** **	** **
SI	0.090	** **	** **	** **	** **	** **	** **	** **
SISO	0.008	** **	** **	** **	** **	** **	** **	** **
UB	** **	** **	** **	** **	** **	** **	** **	** **

## Conclusion, Recommendations, and Limitations

The purpose of conducting this study was to explore factors influencing the acceptance of e-learning systems in higher-educational institutions and its use during the current COVID-19 pandemic. The original constructs from the UTAUT model were used, and the model was extended to measure if Social Isolation influences student's BI of LMS. Furthermore, the extended model also investigated the effect of Corona Fear on the relationship of PE, EE, SI, FC, and Social Isolation with BI of LMS, to analyze how students respond to the technology during the unfortunate emergence of Coronavirus, a highly infectious disease.

The findings show that social isolation, PE, SI, and EE are crucial factors influencing students in Pakistan to pursue the use of LMS while FC does not affect. These results indicate that students will willingly use LMS for successful completion of their courses due to their perception of the benefits provided by the e-learning system, during social distancing. The results concerning moderation of Corona fear revealed that the rise in fear among students regarding the Coronavirus would moderate the relationship of PE and SI on BI of LMS, indicating that students will expect an improved performance by using LMS and will be socially influenced by their friends and family to do so. Moreover, students are not satisfied with the assistance as they consider it difficult to take online classes with poor internet connectivity.

The findings of this study revealed several implications. The first being the extension of the UTAUT model for making it relevant to the current situation caused by the pandemic, and its application in the higher-education sector to investigate the acceptance of e-learning systems. Universities in Pakistan will concentrate on improving student success by enhancing the interface and the learning management system functionality that they introduce. As student's efficiency in learning is increased, they would be motivated to achieve their study goals through the use of LMS, especially when they are socially isolated due to the coronavirus pandemic. Moreover, improving the e-learning system with respect to the effort needed to be invested in using LMS should be a priority as students would be more inclined towards adopting the technology if they perceive it as easy and beneficial to use. Hence, the advantage of using LMS in pandemic when institutions are closed is that it will make students flexible in the future as well. Students will adopt the concept of online education, even when educational institutions will be opened. Therefore, it will provide a great opportunity to educational institutions in terms of earnings and high revenue as they might initiate online courses along with regular classes. So, it is recommended to the management of higher education to establish a strong online portal through which teachers can teach students without any hurdles. Also, the facilitating unit, such as the IT team and student affairs department, has to be efficient enough that they can attract students towards online education for a long term period. Thus, it is suggested to start online diplomas, short-courses, international courses, and regular courses as well. Many students pursue studies with full-time jobs so, it is recommended to initiate online courses for such students. The other advantage is that it enables a centralized pool of information. LMS allows us to keep all information in one single location, and students can access them anytime, anywhere from different locations using compatible devices. This cuts down administrative hassles associated with maintaining learning materials in multiple places. Hence, it saves the cost of educational institutions. It is recommended to work on promoting the use of LMS through successful strategy implementation that will help students analyze the benefits of the technology rather than being intimidated by the change. Furthermore, the adoption of LMS in education is evidence that other educational activities can be done through an online platform. Hence, it is suggested to spread the roots of the online environment and start practicing other activities as well. The world is rapidly shifting towards artificial intelligence, so it's high time to adopt the online environment in our education system.

Limitations of this paper are important to discuss as they hold ground for future research. First of all, a limitation of this paper is the sample size used that can be enlarged to achieve more generalizable results. The sample only consisted of students enrolled in Pakistani universities, which can be further explored by researchers concerning the acceptance of LMS by targeting the faculty of the university, or even through the implementation of a gender-specific study that analyzes and compares behaviors of male and female respondents. Moreover, the authors suggest the need to investigate the extended UTAUT model in other developed and developing countries during the pandemic, to analyze what factors influence acceptance of LMS and use of e-learning systems, to better provide course material and assistance to students in pursuit of education. Other than that, moderating and mediating variables can also be added to extend the model further and evaluate mechanisms relevant to the current situation.
